# Dynamic coronary roadmapping during percutaneous coronary intervention: a feasibility study

**DOI:** 10.1186/s40001-018-0333-x

**Published:** 2018-07-31

**Authors:** Kerstin Piayda, Laura Kleinebrecht, Shazia Afzal, Roland Bullens, Iris ter Horst, Amin Polzin, Verena Veulemans, Lisa Dannenberg, Anna Christina Wimmer, Christian Jung, Florian Bönner, Malte Kelm, Katharina Hellhammer, Tobias Zeus

**Affiliations:** 1 Medical Faculty, Division of Cardiology, Pulmonology and Vascular Medicine, Heinrich-Heine University, Moorenstr. 5, 40225 Düsseldorf, Germany; 2 Philips Healthcare, Eindhoven, Netherlands; 3 CARID (Cardiovascular Research Institute Düsseldorf), Moorenstraße 5, 40225 Düsseldorf, Germany

**Keywords:** Percutaneous coronary intervention, Dynamic coronary roadmap, Real-time overlay, Imaging guided technique

## Abstract

**Background:**

A novel software (“Dynamic Coronary Roadmap”) was developed, which offers a real-time, dynamic overlay of the coronary tree on fluoroscopy. Once the roadmap has been automatically generated during angiography it can be used for navigation during percutaneous coronary interventions (PCI). As a feasibility study, we aimed to investigate the feasibility of real-time dynamic coronary roadmapping and consecutive coronary overlay during elective PCI.

**Methods and results:**

We studied 936 overlay runs, created following the same amount of angiographies, which were generated during 36 PCIs. Feasibility of dynamic coronary roadmapping was analyzed using a dedicated software tool. Roadmap quality (correct dynamic imaging of the vessels without relevant artefacts or missing parts) was distinguished from overlay quality (congruence of dynamic coronary roadmapping and coronary anatomy). Additionally, we assessed procedural success and the occurrence of major cardiac and cerebrovascular events (MACCE). Roadmap quality was defined as “fit for use” in 99.5%. In 97.4% of runs overlay quality was deemed “fit for use”. Overall, we observed low inter and intra observer variability (ICC *R* = 0.84 for roadmap quality and *R* = 0.75 for overlay quality). Procedural success rate was 100%. MACCE occurred in two (5.6%) patients during post-interventional in-hospital stay and were not software-related.

**Conclusions:**

Dynamic coronary roadmapping provides in > 98% of cases sufficient roadmap quality with an anatomically correct overlay of the coronary vessels with good inter and intra observer variability. Future randomized studies are warranted to test possible advantages like procedure time reduction and less consumption of contrast medium.

**Electronic supplementary material:**

The online version of this article (10.1186/s40001-018-0333-x) contains supplementary material, which is available to authorized users.

## Background

Coronary angiography and percutaneous coronary intervention (PCI) are considered as safe and effective procedures with an overall intraprocedural complication rate of 2% [1, 2]. However, some risks like radiation exposure and kidney failure due to contrast medium administration still remain. Acute kidney failure occurs in up to 30% of patients with pre-existing renal impairment [3]. Noteworthy, procedure time, radiation dose and the amount of contrast medium are considerably increased during PCI if compared to diagnostic angiography.

While substantial efforts have been made to improve and develop medical devices such as stents and catheters, the recording technique itself has not been a focus of interest in recent years. Within the past three decades, reduction of radiation exposure was mostly achieved through pulsed imaging and the changeover from analogue to digital recording [4].

The amount of contrast agent necessary for coronary diagnostics and PCI depends on multiple factors: first, and most important, the interventionalists experience, second, biplane or rotational imaging [5] and third, procedural complexity. Particularly during PCI, navigation of wires, balloons and stents within the coronary arteries is challenging and the cardiologist has to use repetitive applications of contrast agent to visualize and control the position of the devices.

Assistance can be provided through a static roadmap of a contrast-filled coronary tree, which is displayed on a second screen. However, this presentation only offers limited support in dynamic settings with moving landmarks and variable anatomical challenges. Therefore, advanced visualization with real-time overlay may be advantageous to facilitate the procedure and reduce side effects. Static overlay techniques already exist for peripheral artery interventions and significantly decrease the applied amount of contrast medium [6]. Unfortunately, these software algorithms are not suitable for coronary imaging due to constant movement of the organ (heartbeat and breathing motion). Based on a conventional coronary angiogram, a novel software algorithm generates a digital overlay of the vessel, which is superimposed on live fluoroscopic images. The cardiologist can navigate within this dynamic coronary roadmap without further application of contrast agent (Additional file 1). In 2015, we already described the first successful application of this novel software algorithm (PCI-Suite, Philips Healthcare, Eindhoven, Netherlands) in a single patient with a bifurcation lesion and real-time overlay guided concomitant PCI [7]. Since then, further refinements of the software algorithms appeared necessary to enhance applicability.

Hereafter, we present our first-in-man feasibility study with dynamic coronary roadmapping. The following primary endpoints were determined (1) *roadmap quality*: whether real-time dynamic coronary roadmaps can be produced with “fit for use” quality in a real-world setting with different coronary anatomies and (2) *overlay quality*: whether congruence of dynamic coronary roadmapping and coronary anatomy could be achieved, ascertained by precise intracoronary device navigation.

The interventionalists were not allowed to rely on the roadmap solely because of its prototype character. Therefore, the interventional strategy and application of contrast agent were not affected by the software. However, to assess the safety profile and to characterize PCI results we defined procedural success and major adverse cardiovascular and cerebral events (MACCE, incidence of cardiac death, myocardial infarction and cerebrovascular bleeding or ischemia) during hospital stay.

## Methods

### Study design

From January to December 2015, a total of 36 patients underwent diagnostic coronary angiography followed by PCI with the support of dynamic coronary roadmapping. Participants initially presented with non-ST-elevation myocardial infarction (NSTEMI) or stable coronary artery disease (CAD) and signs of ischemia. All patients provided written informed consent for data acquisition and analysis. The study was approved by the local ethics committee (MPG-MO-36) and was conducted in accordance to the Declaration of Helsinki. At the time of investigation, the software was used as an additional tool only. The performing cardiologist did not change the interventional approach regarding the image acquisition or the application of contrast agent because safety and feasibility of the software had not been proven in relevant numbers yet. Patient characteristics, periprocedural data and in-hospital data were collected and analyzed.

### Software specifications and application

**Fig. 1:**
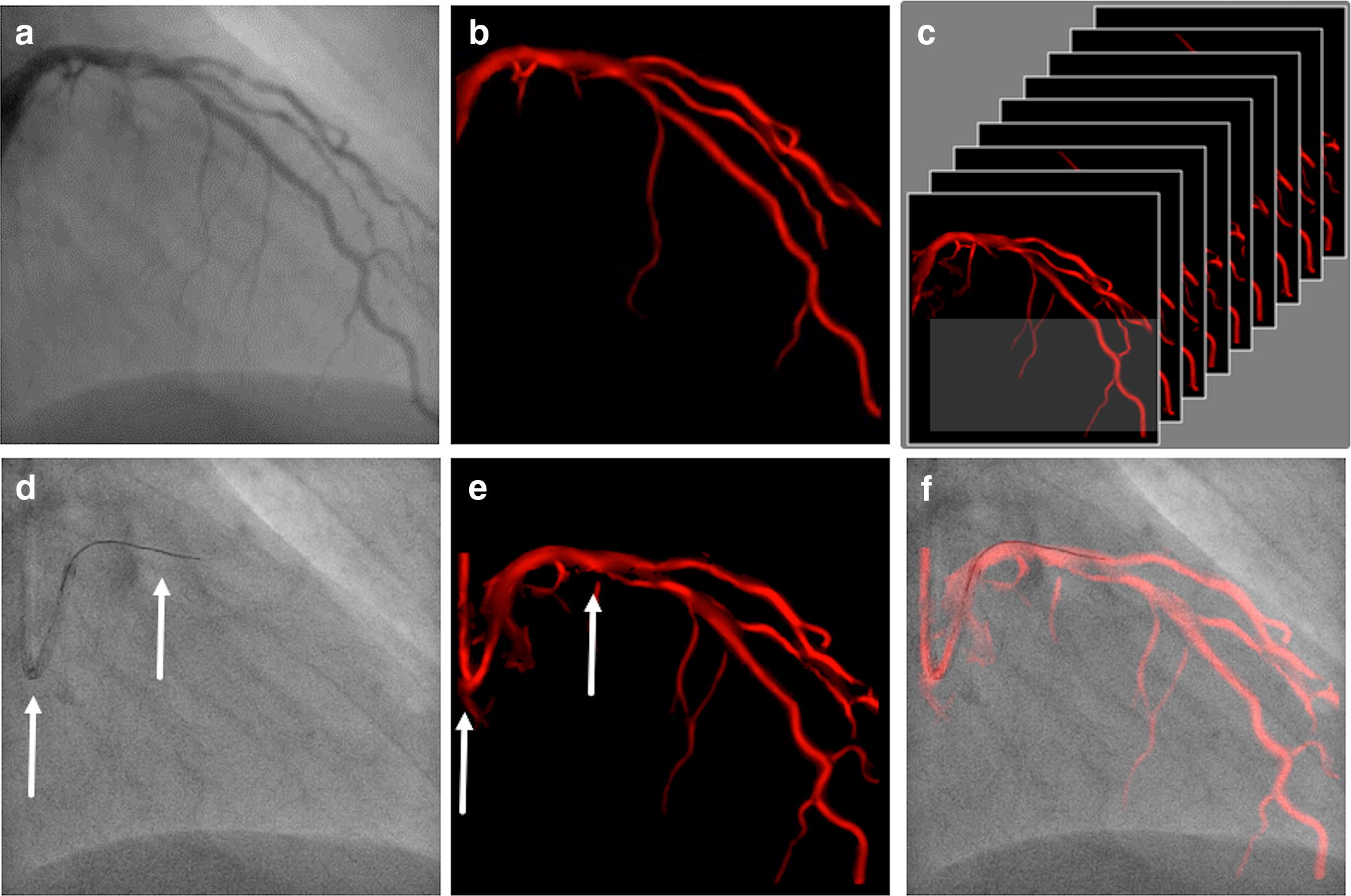
Cardiac roadmapping technology. **a** Cine loop is analyzed on contrast density. **b** Angiography is converted into a mask. **c** A heart cycle of masks is stored in a library, each one defined by its C-arm position. **d** If a corresponding C-arm position is accomplished, the software analyses guide catheter and wire shape (fixed curvatures, arrows). **e** Library search for mask with similar fixed curvatures (arrows) and **f** Superimposed real-time image of the mask and the fluoroscopy image through attachment of the coronary tree to fixed curvatures

**Fig. 2:**
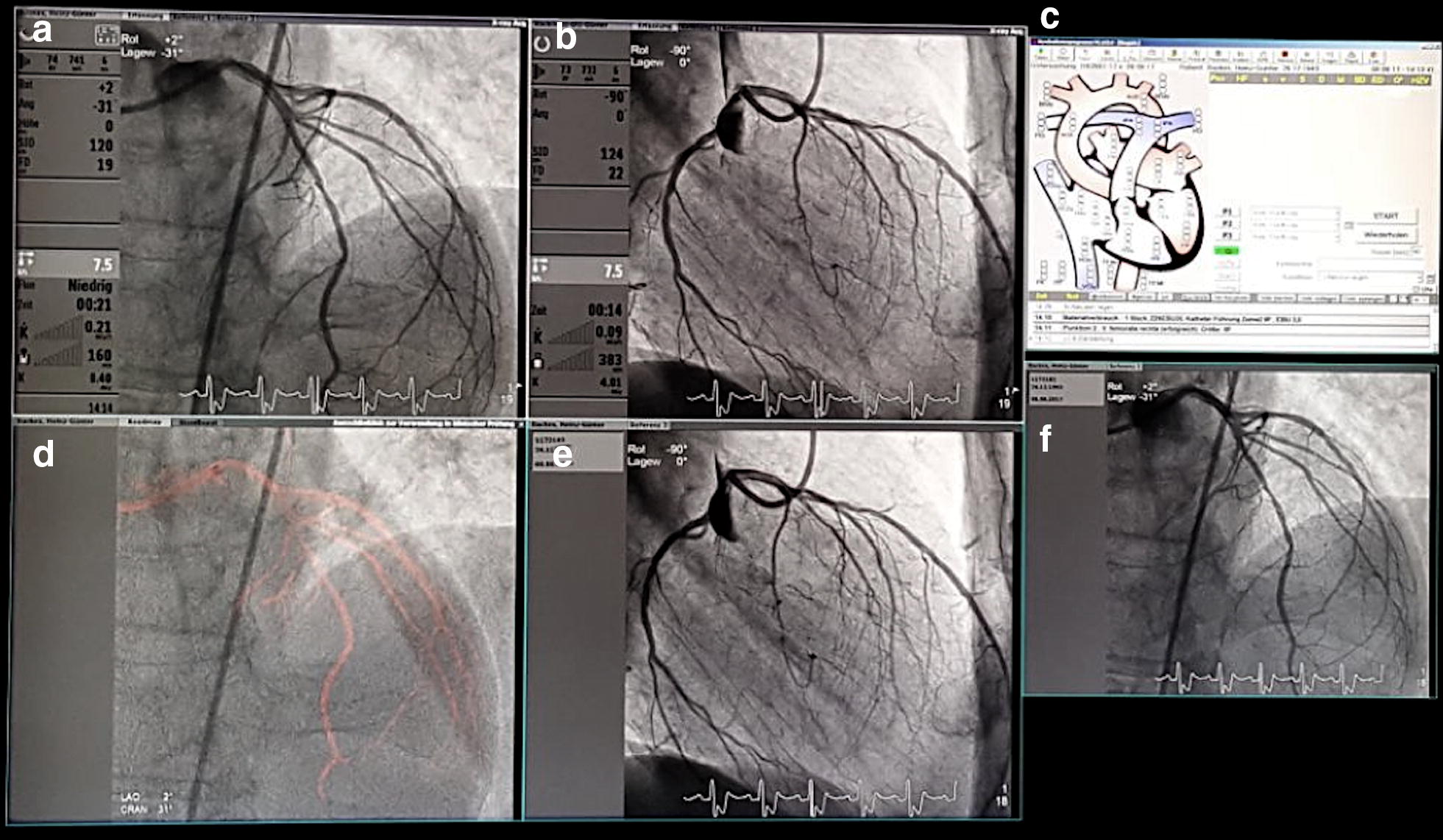
Simultaneous display of dynamic coronary roadmap, static coronary roadmap and standard angiography. **a** Dual plane Angio A, **b** dual plane Angio B, **c** hemodynamics, **d** dynamic coronary roadmap, **e** reference B, **f** reference A

The software was developed and supplied by Philips Healthcare (Best, Netherlands) and analyzed in the context of a master research agreement. To obtain optimal road map images the pulsed image frequency should be at least set to 7.5 pictures per second. This reflects the standard setting of last generation X-ray generators. A roadmap image was automatically generated each time the interventionalist created a cine loop of angiographic images with completely contrast agent filled coronary arteries. Whether contrast was administered by a semi-automatic pump or by hand injection did not influence roadmap quality as long as the coronary tree was completely filled for three cardiac cycles. The amount of contrast ranged from 3 ml for a small right coronary artery to 7 ml for a dominant left coronary artery. Contrast density, guiding catheter and wire shape (if in place) were analyzed by the software and the information converted into a mask. Every time a cine loop with sufficient contrast depiction of the coronary arteries in the same or a different C-arm angulation was recorded, a new mask was produced and converted into a roadmap. Those roadmaps, corresponding to the different C-arm angulations, were stored in a library and automatically presented to the interventionalist as soon as the exact stored C-arm position was obtained again, i.e., during PCI (Fig. 1). Hence the last recorded coronary road map run was displayed on the main screen providing a fuse mask with the actual fluoroscopic image in real time overlay to visualize anatomical conditions and to guide further interventional steps. The software refers to fixed curvatures, it recognizes for example, the guiding or diagnostic catheter and attaches the stored dynamic roadmap to the tip of the catheter. An example of the coronary roadmap displayed together with the standard angiograms on one screen is shown in Fig. 2.

### Definitions

#### Primary endpoint: feasibility

Feasibility was defined as (1) roadmap quality and (2) overlay quality.

1. Road map quality: Every dynamic coronary roadmap, which was stored in the library, was evaluated and graded in “good”, “acceptable” and “suboptimal”. Detailed description of the underlying image characteristics are shown in Table 1. In general, correct imaging of the vessels without relevant artefacts or missing parts was defined as “good”.

**Table 1:** Classification of roadmap and overlay quality

Roadmap quality		
Fit for use	Good	Roadmap shows all relevant vessels, no clutter from background structures
	Acceptable	Roadmap shows some missing parts of the vessel but still gives an impression on the course of the vessels; some clutter in the background but not hindering the image
Insufficient	Suboptimal	Roadmap does not show relevant parts of the vessel, too much clutter in the background
Overlay quality		
Fit for use	Good	All or almost all frames are correctly registered, always clear in which vessel the device is being navigated
	Acceptable	Some frames are not correctly registered but it is always clear in which vessel the device is being navigated
Insufficient	Suboptimal	Registration is not correct, not clear in which vessel the device is being navigated
2. Overlay quality: Every dynamic coronary roadmap, which was shown and automatically recorded during PCI was evaluated and graded in “good”, “acceptable” and “suboptimal”. Detailed description of the underlying image characteristics are shown in Table 1. In general, congruence of dynamic coronary roadmapping and coronary anatomy with intracoronary devices was defined as “good”.

Both, the roadmap and overlay quality were evaluated by three independent, blinded and specifically trained investigators with a purpose-built software (“Cardiac Roadmap”, Philips, Eindhoven). Those reviewed each run and classified the roadmap and overlay quality separately. “Good” and “acceptable” were taken together as “fit for use” because this image quality allows application of the software to guide further interventional steps. Inter observer variability was calculated to rule out information bias.

#### Adverse events and procedural outcome

Procedural success was achieved if stent implantation with grade 3 TIMI flow could be documented. Additionally, MACCE during hospital stay were assessed.

### Statistical methods

Continuous variables were evaluated for normal distribution by Kolmogorov–Smirnov-test and reported as mean with standard deviation. Categorical variables are displayed as percentages of a whole (%). Inter observer variability was calculated by the two-way mixed model with absolute agreement intra class correlation coefficient (ICC). Data analysis was performed using SPSS (SPSS Inc., Chicago, Illinois, USA).

## Results

A total of 36 patients underwent diagnostic coronary angiography directly followed by PCI using dynamic coronary roadmapping. A total of 28 (78.2%) patients presented with NSTEMI, whereas 8 (22.2%) patients were admitted due to CAD with signs of ischemia. A history of previous coronary heart disease was present in all participants. Patient characteristics are shown in the Additional file 2.

### Primary endpoint: feasibility

The cine loop angiographic acquisitions during diagnostic and interventional fluoroscopy resulted in 936 automatically generated coronary roadmaps (on average 26 angiographies per procedure).

1. Roadmap quality: The quality of the roadmap was classified as good in 71.1% (665 runs), acceptable in 28.4% (266 runs) and suboptimal in 0.5% (5 runs) of all cases. Therefore, “fit for use” quality was reached in 99.5%.
2. Overlay quality: The overlay quality was assessed as good in 73.9% (693 runs), acceptable in 23.5% (220 runs) and suboptimal in 2.6% (24 runs) of all images. “Fit for use” overlay quality was achieved in 97.4%.

Overall, we observed low inter and intra observer variability (ICC *R* = 0.84, 95% CI [0.81–0.87] for roadmap quality and *R* = 0.75, 95% CI [0.70–0.79] for overlay quality).

### Examples of insufficient roadmaps or overlays

**Fig. 3:**
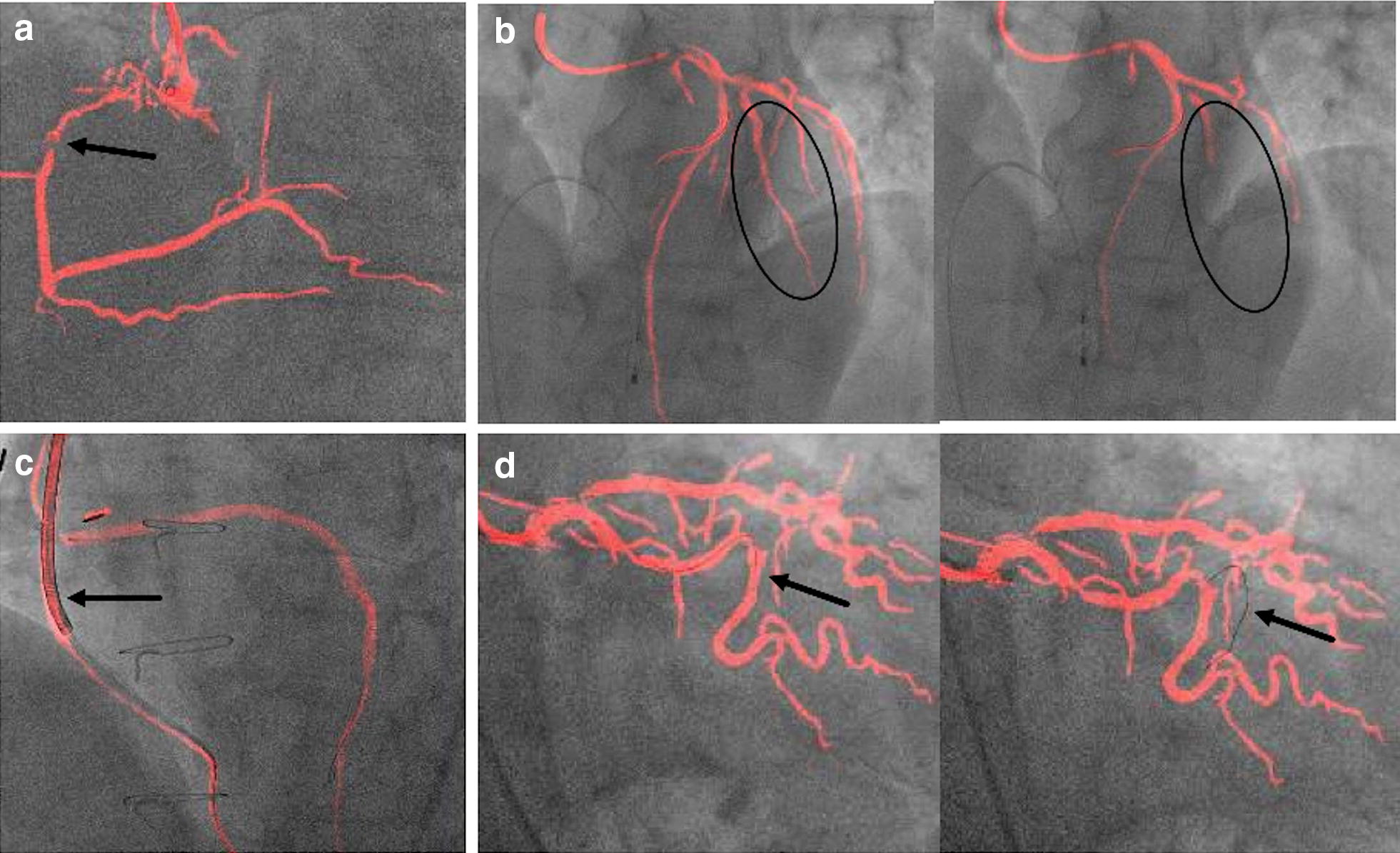
Examples of insufficient roadmap and overlay quality. **a** “Hole“in the map: Some parts of the vessel tree are missing from the roadmap (arrow). **b** “Blinking“vessel: The coronary artery is displayed discontinuously(circles). **c** False device tracking: an ICD lead is falsely displayed as a vessel (arrow). **d** “Jumpy“registration: Exact overlay of the wire and the coronary artery and in the next heartbeat the wire “jumps” and is now displayed next to the vessel (arrows)

Insufficient roadmaps mostly occurred during the process of the intervention when a small or diluted amount of contrast medium was applied, e.g., after application of intracoronary nitrates. As the software always refers to the last recorded run, roadmaps derived from these angiographies were of diminished quality. A phenomenon also occurred that was referred to as a “hole” in the map, when parts of the vessel tree were missing from the roadmap (Fig. 3a). A discontinuous display of the coronary arteries was referred to as “blinking vessels” (Fig. 3b). In some cases, the software mistook devices (pacemaker, cerclages or the guiding catheter) for vessels (Fig. 3c). Occurrence of these errors during real-time coronary roadmap imaging did not always render the roadmap “insufficient” if the area of interest was adequately represented and if the error was not distracting. In some cases, extra beats led to a delay in mask movement and the so-called “jumpy registration” (Fig. 3d), indicating that the wire or catheter did not appear correctly within the overlay. As this phenomenon appears for only a single beat, it does not significantly interfere with roadmap or fusion quality.

### Adverse events and procedural outcomes

Mean procedure time was 58.2 ± 24.1 min and an average of 157.8 ± 70.1 ml contrast agent was applied. Mean fluoroscopy time summed up to 15.3 ± 8 min. The number of implanted stents during one PCI ranged from 1 to 4 with a mean number of 1.8 ± 0.9 stents. In 69.4% of patients, the target lesion was one vessel, whereas in 30.6% of all cases a PCI was performed in two vessels. All stents were implanted successfully with a grade 3 TIMI flow afterwards. An overview of procedural data can be obtained in the Additional file 2.

MACCE occurred in two (5.6%) patients with an increased risk profile and type B2 coronary complexity lesion (according to AHA/ACC lesion classification): One patient presented with a ST-elevation myocardial infarction (STEMI) one day after intervention and received another successful PCI. Patient history included coronary artery bypass grafting, multiple PCIs and a poor left ventricular function. The other patient suffered from cardiac arrest  seven days after the procedure with unsuccessful resuscitation. The relatives declined autopsy, and therefore, the etiology could not be fully clarified. She initially presented with a poor ejection fraction possibly due to tachycardiomyopathy with atrial fibrillation and previous multiple vessel PCI.

## Discussion

The findings of this first-in-man feasibility study with a novel prototype support the concept of dynamic coronary roadmapping. Since this advanced visualization technique has not been evaluated before, categories and parameters to define feasibility were initially developed. Major findings of our prototype study are as follows: (1) the application of dynamic coronary roadmapping during coronary angiography and PCI is feasible. (2) Road map quality and overlay quality are fit for use in almost all (> 98%) runs. (3) Inter and Intra observer variability are good.

This new software tool was designed to enable dynamic coronary roadmapping in a moving organ analogous to the static roadmap overlay in peripheral artery interventions. Potential benefits could be the reduction of the amount of applied contrast medium, procedure duration and dose area product. At this development phase, technical feasibility is the major issue of this application. According to new established and precisely defined criteria, dynamic coronary roadmapping is feasible in our first-in-man study. Another important aspect is that no additional contrast medium has to be administered to create dynamic coronary roadmaps. The automatic and autonomous production and storage of roadmaps makes the application intuitive and simple. Analyzing a new imaging tool the comparison of several blinded investigators is mandatory. We asked three independent specialists to review the runs. Overall low inter and intra observer variability for roadmap quality and for overlay quality could be reported.

To characterize the patient cohort and the procedures entirely, we reported adverse events and procedural characteristics. The incidence of adverse events was relatively high when compared to larger PCI trials. However, these results may be attributed to the relatively small number of patients included in the study. Reported MACCE (one cardiac arrest, one STEMI) could not be associated with the novel software as the investigators worked with standard fluoroscopy images and the dynamic coronary roadmap in a split screen configuration. Moreover, due to its prototype status the interventionalists were not allowed to rely on the software solely.

From the technical perspective it should be mentioned that the software only works on last generation high end X-ray systems. If a biplane angiography unit is used, the roadmap acquisition is only performed with the frontal plane.

## Conclusion

Dynamic coronary roadmapping is feasible during coronary angiography and PCI. It provides “fit for use” roadmap quality and overlay quality in almost all (> 98%) cases. This new imaging technique may have a great potential to improve PCI outcome in the future and based on our encouraging data, prospective trials should be conducted to assess efficacy endpoints.

## Additional files

**Additional file 1.**. Cine loop 1: Wire navigation within the dynamic coronary roadmap without further application of contrast agent.

**Additional file 2.**. **Table S1**. Baseline characteristics and procedural data of patients undergoing PCI with the use of dynamic coronary roadmapping.

## Abbreviations

AHA/ACC: American heart association/American College of Cardiology
CABG: coronary artery bypass graft
CAD: coronary artery disease
CRAN: cranial
DSA: digital subtraction angiography
GFR: glomerular filtration rate
ICC: intra class correlation coefficient
LAD: left anterior descending
LAO: left anterior oblique
NSTEMI: non-ST-elevation myocardial infarction
MACCE: major adverse cardiovascular and cerebral events
PCI: percutaneous coronary intervention
STEMI: ST-elevation myocardial infarction
TIMI: thrombolysis in myocardial infarction
