# Supplementary material for: Dynamic coronary roadmapping during percutaneous coronary intervention: a feasibility study
Source: Eur J Med Res. 2018 Jul 31;23:36. doi: 10.1186/s40001-018-0333-x (PMC6069549; doi:10.1186/s40001-018-0333-x)
Supplement: Supplementary file 2 — Additional file 2.. Table S1. Baseline characteristics and procedural data of patients undergoing PCI with the use of dynamic coronary roadmapping. [file 40001_2018_333_MOESM2_ESM.docx]

**Additional file 2: Table S1.** Baseline characteristics and procedural data of patients undergoing PCI with the use of dynamic coronary roadmapping

| **Baseline** | |
| --- | --- |
| Male | 72.2% (26) |
| Age (years) | 72 ± 11.8 |
| CAD^1^ | 100% (36) |
| Arterial Hypertension | 86.1% (31) |
| Hyperlipidemia | 44.4% (16) |
| Body Mass Index | 26.6 ± 3.8 |
| Smoker | 27.8% (10) |
| Diabetes mellitus | 27.8% (10) |
| Atrial fibrillation | 30.5% (11) |
| Chronic Obstructive Pulmonary Disease | 2.7% (1) |
| LV-Function   - Good - Moderate - Poor | 61.1% (22) 25% (9) 13.9% (5) |
| Previous cardiac surgery   - Coronary Artery Bypass Grafting | 22.2% (8)   - 19.4% (7) |
| Pacemaker | 22.2% (8) |
| GFR^2^ (ml/min/1.73cm^2^) | 68 ± 19.2 |
| Hemoglobin (g/dl) | 11.2 ± 4.2 |
| Reason for admission   - NSTEMI^3^ - CAD with signs of ischemia | 77.8% (28) 22.2% (8) |
| **Procedural data** | |
| Fluoroscopy time (min) | 15.3 ± 8 |
| Mean amount of contrast agent (ml) | 157.8 ± 70.1 |
| Dosage area product (cGy*cm²) | 6786.3 ± 4384.2 |
| Procedure time (min) | 58.2 ± 24.1 |
| PCI^4^, one lesion | 69.4% (25) |
| PCI, two lesions | 30.6% (11) |
| Procedural success | 100% (36) |
| Mean number of implanted stents | 1.8 ± 0.9 |
| Intraprocedural adverse events | 0% |
| Lesion type complexity (according to AHA/ACC^5^ lesion classification)   - A - B1 - B2 - C | - 30.6% (11) - 25% (9) - 35.1% (13) - 8.3% (3) |

^1^CAD = coronary artery disease; ^2^GFR = glomerular filtration rate; ^3^NSTEMI = non-ST-elevation myocardial infarction; ^4^PCI=percutaneous coronary intervention, ^5^ American heart association /American college of cardiology
